# On the origins of endothermy in amniotes

**DOI:** 10.1016/j.isci.2024.109375

**Published:** 2024-03-02

**Authors:** Mathieu G. Faure-Brac, Holly N. Woodward, Paul Aubier, Jorge Cubo

**Affiliations:** 1 Naturhistorisk museum, Univeristetet i Oslo, Sars' gate 1, 0562 Oslo, Norway; 2 Department of Anatomy and Cell Biology, Oklahoma State University Center for Health Sciences, 1111 W. 17th Street, Tulsa, OK 74107, USA; 3 Sorbonne Université, Muséum national d’Histoire naturelle, CNRS, Centre de Recherche en Paléontologie – Paris (CR2P – UMR 7207), 4 place Jussieu, 75005 Paris, France

**Keywords:** Evolutionary biology, Phylogenetics, Phylogeny

## Abstract

A recent study showed evidence that endothermy was ancestral for amniotes using a variety of proxies and a large sample of taxa. However, it did not include numerous crucial taxa. We reevaluated this hypothesis using a large sample of early amniotes and tetrapodomorphs. We inferred the probability of endothermy for each taxon using a model constructed through phylogenetic logistic regressions and using the size of their bone vascular cavities. An ancestral state reconstruction, based on these inferences, was performed to assess the probability of an ancestral endothermy at the node Amniota. Most outgroups were recovered as ectothermic, as is the node Amniota. Our results contradict the hypothesis of an ancestral endothermy and support several independent acquisitions. We discuss that endothermy should be regarded as a collection of acquisitions forming an “endothermic engine” and that studies aimed at inferring endothermy should consider as many of these features as possible.

## Highlights

• Most extinct taxa closely related to the node Amniota have been inferred ectothermic
• Ectothermy is the most probable ancestral state for the clade Amniota
• Endothermy is a sum of many adaptations rather than a unique feature

## Graphical abstract

**Figure:**
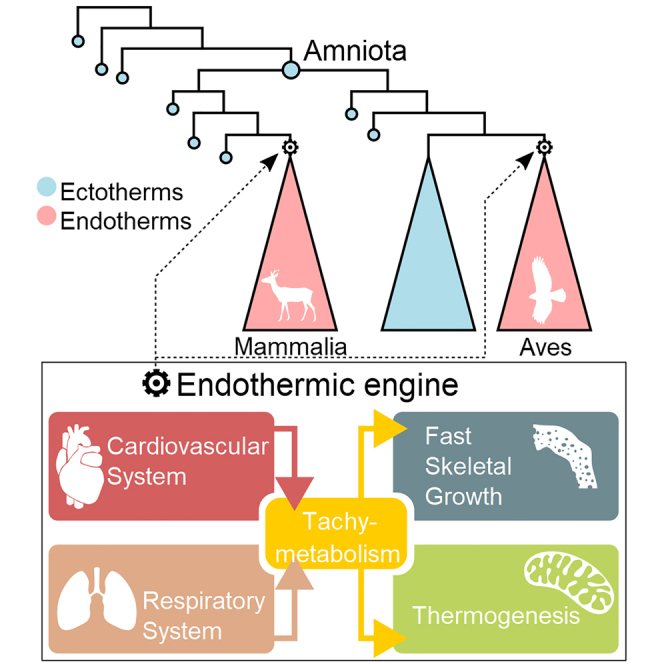

## Introduction

‘Tachymetabolic endothermy’,^4^ the capacity to generate internal heat through metabolic pathways of non-shivering thermogenesis (NST)^1^^,^^2^^,^^3^ at the level of the entire organism, is a type of thermophysiology present in mammals and birds. This feature differs from the ‘regional endothermy’ present in several teleosteans (e.g., opahs, *Lampris* sp.^5^) in that the latter are able to produce internal heat through NST regionally, but do not achieve tachymetabolism (i.e., a high metabolic rate).^1^ This definition also excludes organisms achieving relatively high body temperature (T_b_) through other mechanisms which do not require NST (e.g., gigantothermy, see^6^ for a review). Therefore, hereinafter ‘endothermy’ means ‘tachymetabolic endothermy’.

In living organisms, thermophysiology can be assessed by measuring a specimen’s resting metabolic rate (RMR, i.e., the rate of consumption of oxygen per unit of time during the post-absorptive period) linearly correlated to its expense, and/or its T_b_. Organisms are considered endothermic when their T_b_ is, on average, above 35°C^7^ and their resting metabolic rate is superior to that of the lowest published RMR for extant endotherms (recorded from *Microcebus murinus*, at 1.526 mL(O_2_).h^−1^.g^−0.67^^8^).

Extant birds and mammals, the only extant endothermic amniotes, are crown groups within, respectively, the Sauropsid and Synapsid clades and diverged from a common amniote ancestor over 320 million years ago. For more than two decades, many studies tried to decipher the evolutionary pathways of endothermy within the clade Amniota, using various proxies, including qualitative histology,^9^^,^^10^^,^^11^ quantitative histology,^12^^,^^13^^,^^14^^,^^15^^,^^16^^,^^17^ isotopic geochemistry,^18^^,^^19^^,^^20^ correlation of metabolic rates with body mass,^21^ inner ear biomechanics,^22^ estimation of blood pressure,^23^^,^^24^ body mass growth curves,^25^ or estimation of maximum metabolic rate.^26^ These studies provided evidence that endothermy was present in, at least, four different amniote clades: Archosauromorpha,^27^ Sauropterygia,^14^^,^^20^ Ichthyosauria,^20^ and Therapsida.^13^^,^^28^^,^^29^

A question was then raised: are the archosauromorph, therapsid, ichthyosaurian and sauropterygian endothermies homologous, and, does it therefore constitute a synapomorphy of the clade Amniota? A recent study published by Grigg et al.^4^ addressed that question by reviewing the numerous proxies for endothermy data published in recent years.^4^ They concluded that endothermy was a unique acquisition of the clade Amniota, and, therefore, all its expressions in amniotic species are homologous. However, the quoted study did not consider early amniotes and non-amniote tetrapods phylogenetically and temporally close to the node Amniota, these taxa being supposedly the most representative of the ancestral condition of the clade.

One feature, erythrocyte size, is empirically tightly linked to endothermy,^30^ but has not previously been used to address the question of endothermy at the node Amniota. Here we reevaluate the status of ancestral amniote thermophysiology using (1) early amniote and non-amniote tetrapod taxa and (2) the size of the primary vascular canals, linked to the size of their erythrocytes, as a proxy. Small erythrocytes were identified as a key feature for extant endotherms because their small size and globular shape increases their exchange surface and the reduction (birds) or total removal (mammals) of their nuclear content allow them to deform more easily.^30^ These erythrocyte features permit a higher gas exchange efficiency compared to the larger erythrocytes of ectothermic vertebrates. Such a cell size reduction is associated with a diminution of the capillary diameters, as the erythrocytes must be larger than the capillaries they are passing through in order to deform and increase the oxygen delivery rate.^30^^,^^31^ Thus, the size of bone vascular canals can be used to infer the size of erythrocytes and, through them, the aerobic capacity^17^ and the thermophysiological status^32^ of extinct amniotes. As this feature was not studied by Grigg et al.,^4^ its analysis is an independent test of their ancestral amniote endothermy hypothesis.

The findings of this study contradict partially the results obtained by Grigg et al.^4^: Almost all the species constituting our sample are found ectothermic and ancestral state reconstructions strongly support an ancestral ectothermy in amniotes. We discuss these results and contextualise them in an overall view of the study of endothermy.

## Results

### Quantitative histology

**Figure 1:**
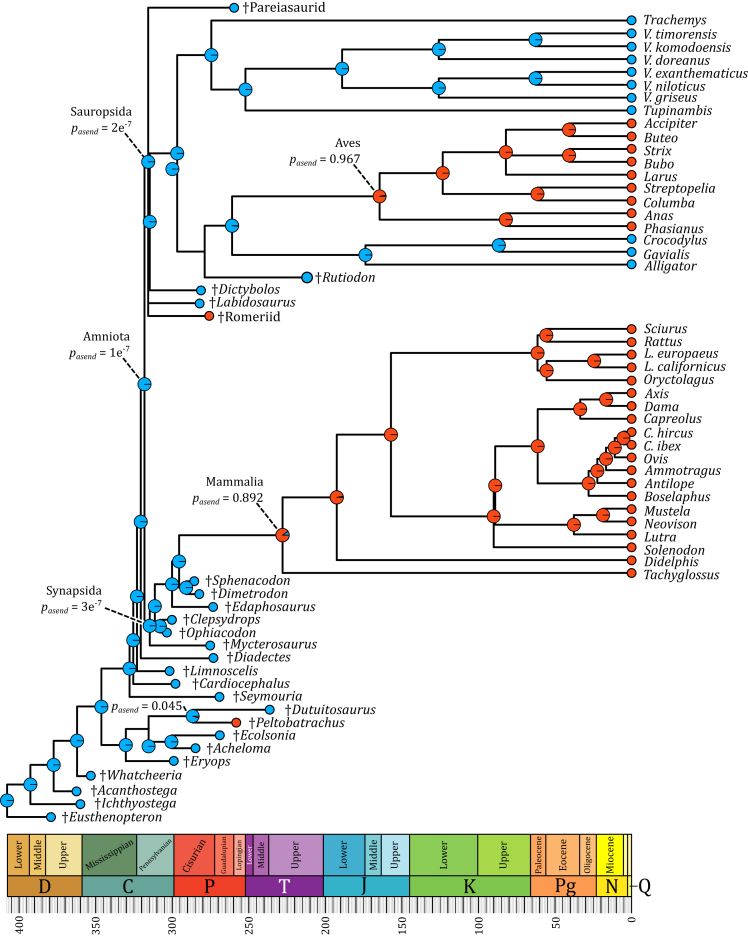
Mean of the 100 inferences of probabilities of ancestral endothermy at each node, using the dating obtained from the ‘equal’ algorithm Blue represents the probability of ancestral ectothermy and red the probability of ancestral endothermy (p_asend_). When the values are not represented, the probability of the most likely regime is superior to 0.96.

**Figure 2:**
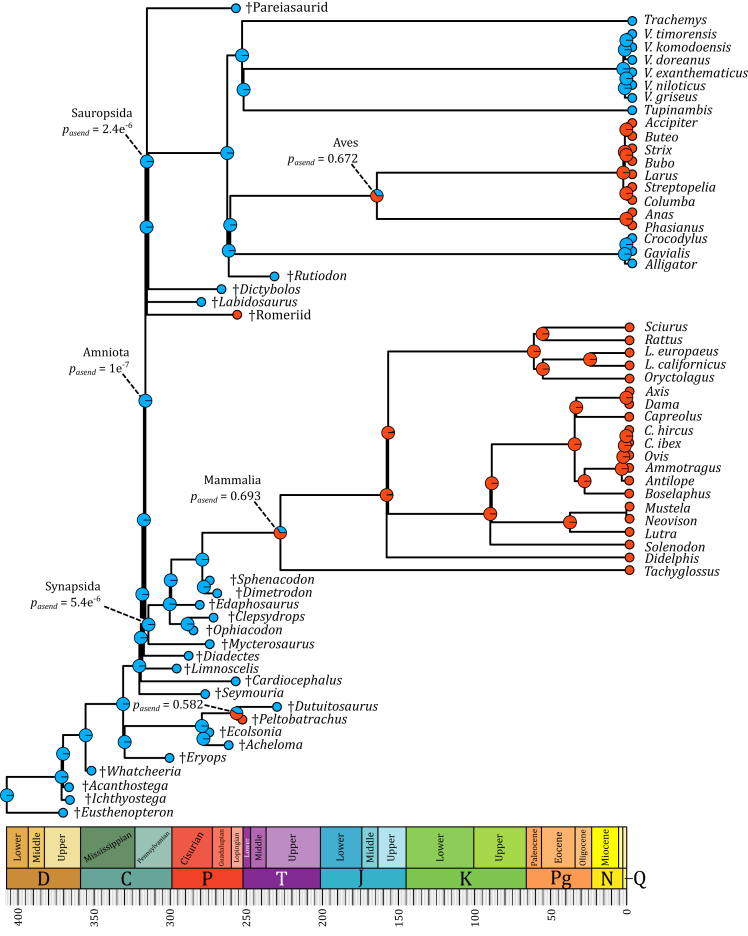
Mean of the 100 inferences of probabilities of ancestral endothermy at each node, using the dating obtained from the ‘mbl’ algorithm Blue represents the probability of ancestral ectothermy and red the probability of ancestral endothermy (p_asend_). When the values are not represented, the probability of the most likely regime is superior to 0.96.

The harmonic mean of the cortical primary vascular canal diameters (HMC), as a proxy for the erythrocyte size, was recorded and computed using Equation 1 (see STAR Methods) for the early amniote taxa included in this study. These values were used to compute a probability for the extinct taxa to have been endothermic ($p_{end}$) using Equation 3 (see STAR Methods). Except for †Romeriid indet. and †*Peltobatrachus*, all extinct taxa in the dataset we used (see Table 1; Figures 1 and 2 for a full list) display high HMC and, consequently, low probabilities of being endothermic, resulting in them being inferred as ectothermic. HMC measurements, $p_{end}$ values, and the inferred thermophysiology for each taxon in this dataset are presented in Table 1.

**Table 1:** HMC, probabilities, and inferred status of the extinct taxa

Taxon	Author	Date	Specimen	HMC	p_end_	Status
†*Acanthostega*	Jarvik	1952	MNHN-Histos-373	29.171	8.35e^−4^	Ectothermy
†*Acheloma*	Cope	1882	MNHN-Histos-375	19.801	5.36e^−2^	Ectothermy
†*Cardiocephalus*	Broili	1904	MNHN-Histos-1890	20.158	4.60e^−2^	Ectothermy
†*Clepsydrops*	Cope	1875	MNHN-Histos-556	16.400	2.07e^−1^	Ectothermy
			MNHN-Histos-556	16.888	1.74e^−1^	Ectothermy
†*Diadectes*	Cope	1878	MNHN-Histos	20.904	3.34e^−2^	Ectothermy
†*Dictybolos*	Olson	1970	MNHN-Histos	29.215	8.19e^−4^	Ectothermy
†*Dimetrodon*	Cope	1878	MNHN-Histos-2712	26.778	2.45e^−3^	Ectothermy
			MNHN-Histos-2713	21.363	2.73e^−2^	Ectothermy
†*Dutuitosaurus*	Hunt	1993	MNHN-Histos-384	28.417	1.17e^−3^	Ectothermy
			MNHN-Histos-386	20.257	4.41e^−2^	Ectothermy
			MNHN-Histos-3060	33.086	1.44e^−4^	Ectothermy
			MNHN-Histos-3068	16.312	2.14e^−1^	Ectothermy
†*Ecolsonia*	Vaughn	1969	MNHN-Histos-376	33.224	2.12e^−4^	Ectothermy
†*Edaphosaurus*	Cope	1882	MNHN-Histos-463	20.060	4.8e^−2^	Ectothermy
†*Eryops*	Cope	1877	MNHN-Histos	25.667	4.03e^−3^	Ectothermy
†*Eusthenopteron*	Whiteaves	1881	MNHN-Histos-531	20.157	4.60e^−2^	Ectothermy
†*Ichthyostega*	Säve-Söderbergh	1932	MNHN-Histos	17.300	1.49e^−1^	Ectothermy
			MNHN-Histos	13.559	4.85e^−1^	Ectothermy
†*Labidosaurus*	Cope	1896	MNHN-Histos-433	25.341	4.66e^−3^	Ectothermy
†*Limnoscelis*	Williston	1911	MNHN-Histos	25.806	3.79e^−3^	Ectothermy
†*Mycterosaurus*	Williston	1915	MNHN-Histos-464	18.211	1.04e^−1^	Ectothermy
†*Ophiacodon*	Marsh	1878	MNHN-Histos-459	19.152	7.06e^−2^	Ectothermy
†Pareiasaurid indet.	–	–	MNHN-Histos	24.920	5.64e^−3^	Ectothermy
†*Peltobatrachus*	Panchen	1959	MNHN-Histos-229	12.136	6.41e^−1^	Endothermy
†Romeriid indet.	–	–	MNHN-Histos-437	10.246	8.07e^−1^	Endothermy
†*Rutiodon*	Emmons	1856	MNHN-Histos-3028	17.413	1.42e^−1^	Ectothermy
†*Seymouria*	Broili	1904	MNHN-Histos-2183	20.607	3.79e^−2^	Ectothermy
			MNHN-Histos-2184	24.190	7.80e^−3^	Ectothermy
†*Sphenacodon*	Marsh	1878	MNHN-Histos-462	23.140	1.25e^−2^	Ectothermy
†*Trematops*	Owen	1859	MNHN-Histos	20.293	4.34e^−2^	Ectothermy
†*Whatcheeria*	Lombard & Bolt	1995	FMNR PR 5022	36.005	3.86e^−5^	Ectothermy
			FMNR PR 5021	36.552	3.02e^−5^	Ectothermy
			FMNR PR 5023	31.221	3.32e^−4^	Ectothermy
			FMNR PR 1962	51.183	4,17e^−8^	Ectothermy

The quantifications of HMC in the fossil genera were obtained using Equation 1, with the resulting probabilities of being endothermic, obtained using Equation 3, and the inferred thermophysiological status. MHNH-Histos refers to section stored at the hard tissue collection of the Muséum national d’Histoire naturelle, Paris, France, and FMNR PR refers to section stored at the hard tissue collection of the Field Museum of Natural History, Chicago, IL, USA. HMC – Harmonic Mean of Canal diameter, in µm; p_end_ – probability of endothermy.

### Ancestral state reconstruction

Ancestral state reconstructions were performed on two sets of 100 trees, with the internal nodes of each set being dated using two different algorithms, named ‘equal’ and ‘mbl’: while the latter scales all zero-length branches to be equal to a specified length (1 Myr in this case), the former projects the nodes without known datum to lie equally between ancestral and derived clades.^33^ At each node, the mean and median value of the probability of ancestral endothermy ($p_{asend}$) was computed. The full procedure is detailed in STAR Methods and in Methods S1. Median and mean values are very close, regardless of the algorithm used for the internal node dating (‘mbl’ or ‘equal’). Therefore, $p_{asend}$ will only refer to the mean of the probabilities of ancestral endothermy of each algorithm hereinafter. Complete numerical results are presented in Results S1. The result of the ‘mean-equal’ analysis is presented in Figure 1 and the result of the ‘mean-mbl’ analysis is presented in Figure 2.

Between the two sets of phylogenies (‘mbl’ and ‘equal’), very little differences of $p_{asend}$ values are found. In both cases, Amniota is inferred as being ancestrally ectothermic with $p_{asend}=1e^{-7}$ (Figures 1 and 2, Results S1). The main difference between these two sets lies in the probabilities associated with the shifts from ectothermy to endothermy in both major clades of endothermic amniotes sampled in our study: archosauromorphs and therapsids (Figures 1 and 2, Results S1). Indeed, while these shifts are inferred to have occurred at the Aves and Mammalia nodes in both sets (Figures 1 and 2, Results S1) the associated $p_{asend}$ values are higher in the ‘equal’ set ($p_{asend}=97\%$ and $p_{asend}=89\%$, Figures 1 and 2, Results S1) than in the ‘mbl’ set ($p_{asend}=67\%$ and $p_{asend}=69\%$, Figures 1 and 2, Results S1). The ancestral state of the clade formed by †*Peltobatrachus* and †*Dutuitosaurus* also differs depending on the internal node dating algorithm. It is inferred as ancestrally ectothermic using the ‘equal’ set ($p_{asend}=4.5\%$, Figures 1 and 2, Results S1) while it is inferred as ancestrally endothermic using the ‘mbl’ set ($p_{end}=58\%$, Figures 1 and 2, Results S1).

## Discussion

Erythrocyte size is, at present, an unequivocal metric to assess thermophysiological status.^17^^,^^30^ Using vascular canal diameter as a proxy for erythrocyte size and, then, endothermy, our results contradict Grigg et al.^4^’s hypothesis of an ancestral endothermy for the clade Amniota. It should be noted that our study does not aim to elucidate the acquisition of endothermy in the clades Synapsida and Archosauromorpha. Therefore, we will not discuss these nodes because our sample is not suited to do so. A reader interested in the timing of the apparition of endothermy in these two clades will find relevant information in other publications.^7^^,^^17^^,^^27^^,^^28^^,^^29^

**Figure 3:**
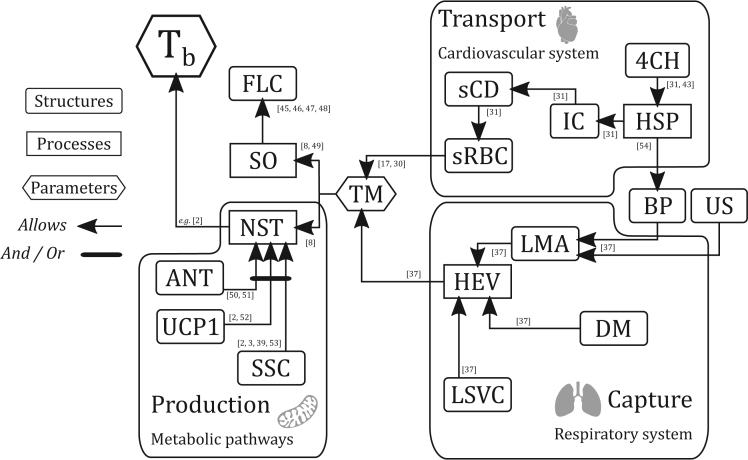
Schematic representation of the “endothermic engine”, i.e., the different adaptations necessary to sustain endothermy in extant endothermic amniotes See the text for a detailed explanation. Abbreviations: 4CH – Four chambered heart; ANT – Adenine Nucleotide Translocator; BP – bipedality; DM – Diaphragmatic muscle; FLC – Fibrolamellar complex; HEV – Highly efficient ventilation; HSP – high systemic pressure; LMA – Locomotor muscles attachments; LSVC – Lateral stability of the vertebral column; NST – Non shivering thermogenesis; sCD – small capillary diameters; SO – Static osteogenesis; sRBC – small erythrocytes; SSC – Sarcolipin-SERCA complex; Tb – Body temperature; TM – Tachymetabolism; UCP1 – Uncoupling protein 1; IC – Increased Capillarization; US – upright stance. The number associated to the arrows directly refers to the references listed at the end of the paper. Additionnal references were used to produce this figure and can be found in the bibliography alongside to the others.^45^^,^^46^^,^^47^^,^^48^^,^^49^^,^^50^^,^^51^^,^^52^^,^^53^^,^^54^

Despite results from our proxy contradicting the proposal of ancestral endothermy in Amniota from Grigg et al.,^4^ the results do not fully reject their hypothesis. While our study focused specifically on the size of erythrocytes, Grigg et al.^4^ used a set of different morpho-anatomical and histological features, such as the presence of fibrolamellar complex, and of a blood pressure diagnostic of a four chambered heart, commonly associated with endothermy. As the resulting conclusions differ depending upon the proxy used, it seems clear that endothermy can hardly be defined as a unique feature, but rather as the acquisition of various features, a kind of “endothermic engine” (Figure 3). Indeed, a collection of implicated parts can be identified in extant endotherms and forms a complex model we describe in the following lines.

Endothermy has been defined as the capacity to generate heat through NST.^1^^,^^2^^,^^4^ Thus, the presence of a routinely working NST pathway, such as a working Sarcolipin-SERCA complex (SSC), is required to conclude that a given species is endothermic. However, in most cases a given species is considered as being endothermic based on the presence of indirect evidence (such as a high RMR^8^). SSC is currently the best candidate to fulfill the role of routinely active NST as it has been demonstrated *in vitro,*^35^ and several studies suggested its role in NST expression *in vivo* in eutherians (see Bal and Periasamy^3^ for a review). However, its activity has still to be shown in many extant endothermic species, especially in birds,^2^^,^^3^ and there are debates about whether SSC is the principal NST pathway or not.^36^ Thus, the presence of the different components of this complex, i.e., sarcolipin and SERCA, is not enough to infer a working SSC as NST, because (1) both molecules exist in ectothermic species^2^ and (2) as exemplified in extant endotherms, having a routinely active NST requires a huge amount of energy: the RMR of an extant endothermic species being generally 10 times higher than that of an ectothermic species.^8^ Therefore, organisms must be able to achieve a very high metabolic rate to fuel SSC. In practice, the proxy most frequently used to show evidence for endothermy is not the presence of molecular components to perform NST, but rather the presence of tachymetabolism.

The metabolic rate measures the capacity of an organism to produce and use energy, based on its oxygen consumption. Therefore, tachymetabolism is associated with efficient capture and transport of oxygen. Extant endothermic species present a collection of dedicated adaptations, such as within the respiratory and cardiovascular systems, which does not exist in ectothermic species and form thus the “endothermic engine” mentioned above (Figure 3). Carrier^37^ identified several features allowing efficient breathing tightly associated with endothermy: the unloading of the respiratory systems thanks to large vertebral processes supporting the locomotor muscles and upright stance or bipedality, the possession of diaphragmatic muscles and the lateral stability of the vertebral column. The unidirectional airflow was also presented as a putative feature contributing to more efficient ventilation,^38^ but it was recently identified in lepidosaurians,^39^^,^^40^ and crocodilians.^40^^,^^41^^,^^42^ While the presence of a unidirectional airflow in Crocodylia could be explained by the inheritance from an endothermic ancestor,^15^^,^^43^ its presence in lepidosaurians prevents unequivocal association with either endothermy or ectothermy and cannot be used to advocate for the evolution of one or the other.^38^ Other structures, such as the presence of air sacs, could potentially be linked to tachymetabolism but more studies are needed to test such hypotheses.

On the other hand, the transport of oxygen through the systemic circulation benefits from adaptations of the cardiovascular system. As explained in the Introduction, a reduction of the size of erythrocytes is linked to endothermy.^17^ This feature highly improves gas exchange and leads to an increased capillarization of tissues because of the associated reduction of the diameter of capillaries but it leads to an increase of the blood flow resistance.^31^ Such resistance cannot be overcome with a low systemic pressure and, therefore, can only be achieved with a full separation of pulmonary and systemic circulations by a four chambered heart.^31^^,^^43^ Considering the model proposed in Figure 3, it is important to stress that the strength of our proxy lies in the inference of a feature involved in the sustainment of tachymetabolism, rather than resulting *from* sustained tachymetabolism, as could be the case for other proxies such as an extensive fibrolamellar complex. Missing this part, as well as any other part implicated in the sustainment of tachymetabolism (respiratory and cardiovascular systems on Figure 3) will, then, contradict the functioning of the engine.

Thus, while small erythrocyte size is a key component, all the adaptations discussed here are necessary to increase the quantity of oxygen fueling the mitochondria in extant endothermic species. Highly efficient engines that are the endotherms cannot work optimally if they do not possess all the required parts. Our study demonstrates that at least one of those parts, small erythrocytes, was missing in the first amniotes. An interesting case study is †*Whatcheeria*. This tetrapodomorph, which lived during the Carboniferous Period, was inferred as being ectothermic in our study, based on canal size. However, it displays FLC in its femoral cross section,^44^ a type of tissue frequently used as evidence for endothermy by numerous studies, including Grigg et al.^4^ However, †*Whatcheeria*’s HMC is in accordance with big erythrocytes, suggesting its cardiovascular system did not attain a sufficient efficiency to sustain tachymetabolism (Figure 3). Based on our model, it is improbable †*Whatcheeria* was able to sustain tachymetabolism, therefore suggesting that the sole presence of FLC is not definitive evidence for endothermy. More studies are needed to infer the presence or the absence of other parts of our model to establish clearly the real thermophysiological status of †*Whatcheeria*.

Finally, we would like to briefly address describing endothermy in terms of homology. As endothermy is the outcome of a combination of several very different components, it is more a function than a structure. Thus, endothermy cannot be qualified as homologous, as homology can only concern structures.^55^ Instead, only the different structural components of endothermy could be homologous, and that has still to be tested. For instance, the acquisition of small erythrocytes was clearly the result of different evolutionary processes: while mammals evolved enucleate erythrocytes, birds lost a great part of their genetic material, leading to the same effect.^30^ Functions being labile, it is not hard to imagine that endothermy appeared and disappeared several times in different clades under different selective pressures, while its structural components are still in place. This kind of scenario probably occurred in the loss of endothermy or, at least, tachymetabolism, in the crocodilian clade.^12^^,^^15^^,^^32^^,^^43^

### Limitations of the study

Differences in the values of $p_{asend}$, the probability of an internal node to have been endothermic, between the two different internal nodes dating algorithms are due to the differences in branch lengths: with the ‘mbl’ computation, the branch length between †*Peltobatrachus*, inferred as endothermic, and its direct more inclusive node is smaller than using the ‘equal’ computation. †*Peltobatrachus’* $p_{end}$ has thus more weight on the probability of the ancestral state than †*Dutuitosaurus*’, whose branch length varies less. The shorter the branch linking a taxon to its closest inclusive node is, the higher the taxon’s $p_{end}$ weights in the estimation of its probable ancestral state. It is thus not surprising that the Amniota clade is inferred as ancestrally ectothermic since it is immediately surrounded by ectothermic taxa (except for the †Romeriid indet.). The same can be said with the estimation of $p_{asend}$ at the nodes Aves and Mammalia. While branch lengths impact these nodes, the estimation of $p_{asend}$ at the node Amniota remains unchanged (<0.0001%). Because the dating of both the species and internal nodes as well as the topology of the tree determine the ancestral state reconstructions, the validity of the latter depends on the reliability of the former. Competing phylogenetic hypotheses as well as the discovery of new fossils could thus render these results obsolete.

Moreover, our model must be taken with caution: as shown in the figure in quantification and statistical analysis, it failed to assign five extant taxa (three ectotherms and two endotherms) an appropriate $p_{end}$ according to the threshold of 0.59. The relationship between the size of the erythrocytes and HMC is thus not always exact, despite the statistical relationship still being significant.^32^ Moreover, Starck and Chinsamy^34^ demonstrated that the size of vascular canals can vary accordingly to the availability of food during osteogenesis: less available food leads to smaller vascular canals and, consequently, to an overestimation of $p_{end}$. Then, an ectothermic taxon can be wrongly associated with endothermy because of food limitation. Thus, this model can only reject endothermy confidently. However, it is perfectly suited to discuss this issue as we try to test the hypothesis supported by Grigg et al.^4^ of an ancestral endothermy.

### Conclusion

Are our results obtained using vascular canal size as a proxy for endothermy enough to reject the hypothesis of ancestral amniote endothermy? The major importance of the size of erythrocytes in the endothermic engine and the sample of studied taxa is, in our opinion, enough to challenge Grigg et al.^4^’s hypothesis, but more studies on taxa closely related to the node Amniota focusing on all components of the endothermic engine are required to answer this question.

Then, do the different extant cases of endothermy (mammal and birds) have one and the same origin? The results of this study contradict a positive answer. As a function, endothermy was probably acquired several times in different clades, such as Archosauriformes,^12^^,^^27^ Therapsids,^13^^,^^28^^,^^29^ Sauropterygia,^14^^,^^20^ and Ichthyosauria.^20^ However, are the different structures involved in NST homologous? Probably yes, for at least some of them, such as the SSC.

Thus, we suggest more caution when dealing with this topic. Endothermy might well have been present in the first amniotes, but there are still many uncertainties to elucidate before reaching such a conclusion.

## STAR★Methods

### Key resources table

**Table:** 

REAGENT or RESOURCE	SOURCE	IDENTIFIER
**Deposited data**		

HMC measurements	This study	Table 1
FAD and LAD	This study	Data S3
Handmade meta-phylogenetic tree	This study	Data S1

**Software and algorithms**		

Fiji	Schindelin et al. (2012)^56^	https://imagej.net/software/fiji/downloads
R - v4.3.0	CRAN^59^	https://cran.r-project.org/
Package ‘phytools’ for R - v1.9-16	Revell et al. (2012)^60^	https://github.com/liamrevell/phytools
Package ‘TreeTools’ for R - v1.10.0	Smith (2019)	https://github.com/ms609/TreeTools/
Package ‘evobiR’ for R - v1.1	Blackmon and Adams (2015)	https://github.com/coleoguy/evobir
Package ‘paleotree’ for R - v3.4.5	Bapst (2012)	https://github.com/dwbapst/paleotree
Script used for R	This study	Data S2

**Others – Osteohistological sections**		

†*Acanthostega*		MNHN-Histos-373
†*Acheloma*		MNHN-Histos-375
†*Cardiocephalus*		MNHN-Histos-1890
†*Clepsydrops*		MNHN-Histos-556
†*Diadectes*		MNHN-Histos, ‘de Ricqles Cabinet’
†*Dictybolos*		MNHN-Histos, ‘de Ricqles Cabinet’
†*Dimetrodon*		MNHN-Histos-2712, 2713
†*Dutuitosaurus*		MNHN-Histos-384, 386, 3060, 3068
†*Ecolsonia*		MNHN-Histos-376
†*Edaphosaurus*		MNHN-Histos-463
†*Eryops*		MNHN-Histos, ‘de Ricqles Cabinet’
†*Eusthenopteron*		MNHN-Histos-531
†*Ichthyostega*		MNHN-Histos, no associated number
†*Labidosaurus*		MNHN-Histos-433
†*Limnoscelis*		MNHN-Histos, ‘de Ricqles Cabinet’
†*Mycterosaurus*		MNHN-Histos-464
†*Ophiacodon*		MNHN-Histos-459
†Pareiasaurid indet.		MNHN-Histos, ‘de Ricqles Cabinet’
†*Peltobatrachus*		MNHN-Histos-229
†Romeriid indet.		MNHN-Histos-437
†*Rutiodon*		MNHN-Histos-3028
†*Seymouria*		MNHN-Histos-2183, 2184
†*Sphenacodon*		MNHN-Histos-462
†*Trematops*		MNHN-Histos, ‘de Ricqles Cabinet’
†*Whatcheeria*		FMNR PR 5021, 5022, 5023

### Resource availability

#### Lead contact

Further information and requests for resources should be directed to and fulfilled by the Lead Contact, Mathieu G. Faure-Brac (faurebrac.mathieu@gmail.com).

#### Materials availability

All the studied specimens (except for †*Whatcheeria*) are stored in hard tissue collection of the Natural History Museum of Paris and are available upon request to its curator, D. Germain.

#### Data and code availability

• The raw dataset, comprising the histological measurements and the probability computed for a sample of 23 extinct tetrapodomorph species, including taxa classified as stem amniotes as well as immediate outgroups, is presented in Table 1.
• The phylogenetic tree, in a Newick format, the code used to produce our analyses, and the first and last appearance data associated to each specimen are available in, respectively, Data S1, S2 and S3.
• Any additional information required to reanalyse the data reported in this paper is available from the lead contact upon request.

### Method details

We used the protocol developed by Cubo et al.^32^ to infer, for each fossil taxon, the probability of having been endothermic, using quantitative osteohistology. Mid-diaphyseal femoral cross sections of extinct tetrapodomorphs were examined. The sample set, consisting of 31 specimens representing 24 genera (except for †*Whatcheeria*), is curated at the Vertebrate Hard Tissue Collection Muséum national d’Histoire naturelle, Paris, and is available upon request to its curator. For each thin section, the entire specimen was imaged through a series of sequentially taken photomicrographs. The image series was then imported into Adobe Photoshop CC, using the Photomerge command. The software aligned the individual images from the input series to produce a composite of the entire thin section. Minimum vascular canal diameters were quantified using the resulting composite images.

To perform our analysis, an informal supertree was compiled. The protocol and sources are detailed in Methods S1. This unique topology comprises all extinct species studied here (Table 1), and all extant ones used in Cubo et al.^32^ Because branch lengths are needed to reconstruct the ancestral states, we used the stratigraphic range of our fossil taxa to compute lengths reflecting the time between each pair of nodes and node-leaves. We gathered the first and last apparition datum published for each taxon from the PaleoBiology Database (last access: April 23rd, 2023, at https://paleobiodb.org). However, these stratigraphic ranges allow uncertainty about the real stratigraphic range of taxa and, subsequently, on the age associated to each internal node. We thus used the function *timePaleoPhy* from the package ’paleotree’^58^ in R v.4.3.0^59^ which samples a random datum in each stratigraphic range and associates an age to each internal node coherent with this sample. Two different algorithms (the ’equal’ and ’mbl’ methods) were used to compute branch lengths, with 100 repetitions for each algorithm.

### Quantification and statistical analysis

We used a methodology adapted and modified from Huttenlocker and Farmer^17^ to sample bone vascular canal diameter. The outlines of primary vascular canals were traced, and vascular canal diameters obtained using the software program Fiji.^56^ However, the present study did not restrict primary vascular canal sampling to the mid- and outer cortex. Instead, whenever possible, we recorded every primary vascular canal observed in the transverse section even when the resulting sample size exceeded one hundred vascular canals. Then we computed the HMC for each specimen using Equation 1:

(Equation 1) $$HMC=\frac{n}{\sum_{i=1}^{n}\left(1/x_{i}\right)}$$

with n the number of quantified canals, and x the values of the diameters.

Cubo et al.^32^ developed a protocol using phylogenetic logistic regression^57^ to infer the probability of being endothermic using HMC as a proxy. This method tests the relationship between the two states (here, endothermy and ectothermy) of a binary response variable (here, the thermophysiology) and different quantitative or discrete explanatory variables (here the harmonic mean of canal diameter) in a phylogenetic context. If significant, it allows using of Equation 2 to compute the probability for an inferred taxon to having been endothermic:

(Equation 2) $$p_{end}=\frac{e^{c\times HMC+i}}{1+e^{c\times HMC+i}}$$

with $p_{end}$ the probability to having been endothermic, c the coefficient associated to the explanatory variable, HMC, and i the intercept. The coefficient and intercept values published by Cubo et al.^32^ were then integrated to Equation 3, which is then applied to our data:

(Equation 3) $$p_{end}=\frac{e^{-0.45\times HMC+6.04}}{1+e^{-0.45\times HMC+6.04}}$$

To perform the ancestral state reconstruction, extinct species needed, firstly, to be assessed as endothermic or ectothermic. The $p_{end}$ value obtained using Equation 3 for each fossil taxon was compared to the cut-off probability determined by Cubo et al.^32^: 0.59 (see below figure). If $p_{end}\geq 0.59$, then the taxon was inferred as endothermic. If $p_{end}<0.59$, then the taxon was inferred as ectothermic.

**Figure:**
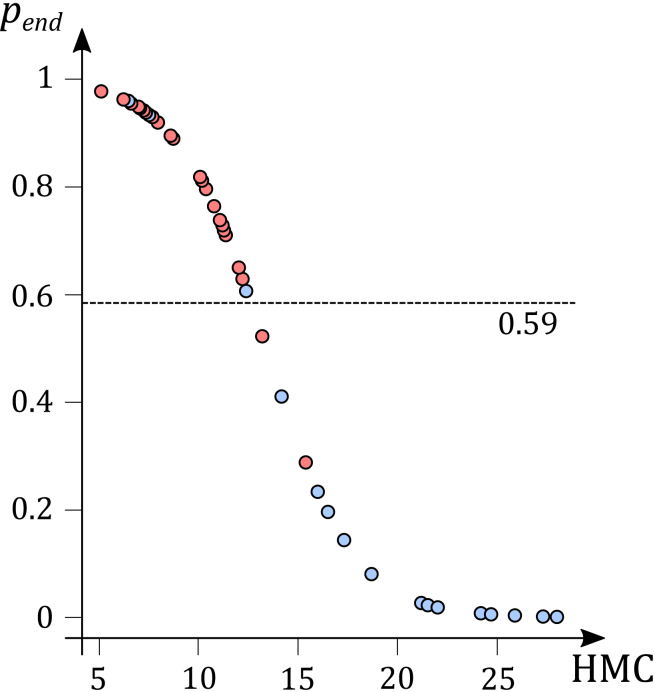
Distribution of probabilities of being endothermic inferred for the sample of extant tetrapods using a phylogenetic logistic regression model that includes HMC as the explanatory variable The red circles correspond to extant endothermic tetrapods, the blue ones to extant ectothermic tetrapods. The dotted line corresponds to the threshold between endothermy and ectothermy according to this model (modified from Figure 3 of Cubo et al.^32^). HMC - Harmonic Mean of of Canal diameter, in µm; p_end_ - probability of endothermy.

The ancestral states reconstruction was performed on our 200 phylogenies, using the function *rerootingMethod* from the package ’phytools’,^60^ in R. v4.3.0^59^. It produces, at each internal node, a probability for this node to have been endothermic or ectothermic. For each two sets of phylogenies (produced using the ’mbl’ or ’equal’ algorithms), we computed the mean and median of the inferred probabilities at each node. Therefore, we produced four analyses: ’mbl-median’, ’mbl-mean’, ’equal-median’ and ’equal-mean’.

## Supplemental information

Document S1. Methods S1: containing Figure S1 and the method used for dating the tree, related to STAR Methods

Data S1. Informal meta-phylogenetic tree, produced accordingly to Methods S1, related to STAR Methods

Data S2. R script used for dating of the tree and the ancestral states reconstructions, related to Figures 2 and 3 and STAR Methods

Data S3. FAD and LAD used for dating the tree, related to STAR Methods

Results S1. mean and median probabilities for each nodes according to the two different dating algorithms, related to Figures 2 and 3
