# Supplementary material for: On the origins of endothermy in amniotes
Source: iScience. 2024 Mar 2;27(4):109375. doi: 10.1016/j.isci.2024.109375 (PMC10966186; doi:10.1016/j.isci.2024.109375)
Supplement: Document S1. Methods S1: containing Figure S1 and the method used for dating the tree, related to STAR Methods [file mmc1.pdf]

iScience, Volume *27*

Supplemental information

On the origins of endothermy in amniotes

Mathieu G. Faure-Brac, Holly N. Woodward, Paul Aubier, and Jorge Cubo

~~Supplementary File 2~~Methods S1

Contents

1. ~~Supplementary Figure~~Figure S~~-~~1 – Phylogenetic tree displaying the number corresponding to each internal node and used in the ~~Supplementary File 5~~Results S1, related to STAR Methods.

2. ~~Supplementary~~ Methods S1– The detailed protocol used to compile the phylogenetic tree displayed in ~~SF1~~Figure S1, related to STAR Methods.

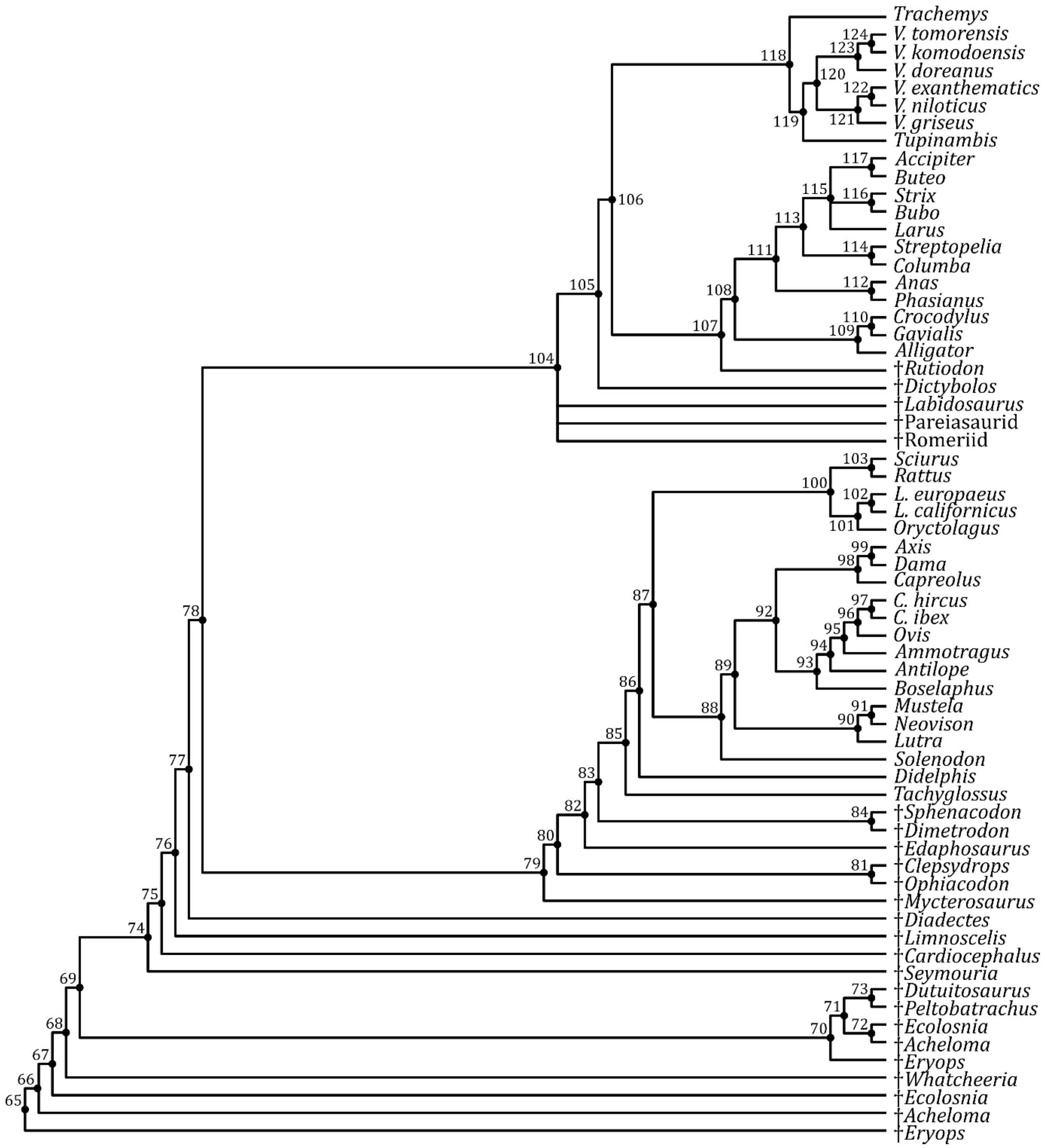

~~Supplementary~~ Figure S1. Phylogenetic tree used in the analyses. This tree is an informal supertree aggregated from different sources. See the protocol detailed in ~~Supplementary Methods~~Methods S1 above to get the full list of the sources. Numbers at each node corresponds to the one used in ~~Supplementary File 5~~Results S1.

2. Supplementary Methods S1

We assembled our tree using the following methodology. Firstly, we compiled a set of species of interest, encompassing the extinct taxa we are studying here and the extant sample from Cubo et al.[1]. As we needed data associated with species registered in the Palaeobiology Database (https://paleobiodb.org/classic), we kept only taxonomically valid species. Thus, *nomen dubium* and *nomen vanum* were discarded. The studied specimens concerned by this taxonomic uncertainty were merged into other valid species whenever it was possible.

Once our list of valid species was established, we built the tree itself. A collection of recent phylogenies was made, chosen to cover the different levels of integration of the final tree. Some trees were chosen because of their focus on the relationships inside clades[2,3,4,5,6], while others were chosen because they focus on the relationships between these clades[7,8,9]. These studies deal only with extinct taxa and the tree from Cubo et al.[1] was used to cover the extant ones. The resulting tree is a handmade compilation considering the potential incongruences. The data source of these trees, *i.e.* morphological, molecular, etc., and the methods used to reconstruct them were not assessed here, as we considered only their topology and the hypotheses of the phylogenetic relationships they convey.

After this step, we collected age data from the Paleobiology Database. First (FAD) and last apparition datum (LAD) were recorded for each operational taxonomic unit included in the phylogeny. In order to obtain the largest possible time interval during which these species potentially existed. Extant taxa received both a LAD and FAD of 0.

Whenever possible, internal nodes and especially the root, were assigned a minimal age of appearance in case it was not fully appreciated by their sampled diversity. If the first appearance datum of a given clade is older than our sample suggests (*e.g.,* Mammalia) then a FAD based on the oldest known record of the clade was assessed manually (*e.g.*, 235 Myr for Mammalia).

Finally, the tree was dated using the function *timePaleoPhy* from the 'PaleoTree' package[10], using R v4.3.0[11]. This function randomized the branch length using the uncertainty of

appearance of extinct taxa through their stratigraphic range, indicated by their LAD and FAD, and using two different algorithms (see main manuscript). The code is provided in ~~Supplementary File~~ Data S2.

~~3.~~References

1. Cubo, J., Aubier, P., Faure-Brac, M. G., Martet, G., Pellarin, R., Pelletan, I., and Sena, M. V. A. (2023). Paleohistological inferences of thermometabolic regimes in Notosuchia (Pseudosuchia: Crocodylomorpha) revisited. Paleobiology 49, 1–11. https://doi.org/10.1017/pab.2022.28.

2. Swartz, B. (2012). A marine stem-tetrapod from the Devonian of western North America. PLoS ONE *7*, e33683. https://doi.org/10.1371/journal.pone.0033683

3. Ezcurra, M. D. (2016). The phylogenetic relationships of basal archosauromorphs, with an emphasis on the systematics of proterosuchian archosauriforms. PeerJ *4*, e1778. https://doi.org/10.7717/peerj.1778

4. Silva-Neves, E., Modesto, S. P., and Dias-da-Silva, S. (2018). A new, nearly complete skull of Procolophon trigoniceps Owen, 1876 from the Sanga do Cabral Supersequence, Lower Triassic of Southern Brazil, with phylogenetic remarks. Historical Biology *32*, 574-582. https://doi.org/10.1080/08912963.2018.1512106

5. Van den Brandt, M. J., Abdala, F., and Rubidge, B. S. (2020). Cranial morphology and phylogenetic relationships of the Middle Permian pareiasaur *Embrithosaurus schwarzi* from the Karoo Basin of South Africa. Zoological Journal of the Linnean Society *188*, 202-241. https://doi.org/10.1093/zoolinnean/zlz064

6. Bickelmann, C., Müller, J., and Reisz, R. R. (2009). The enigmatic diapsid Acerosodontosaurus piveteaui (Reptilia: Neodiapsida) from the Upper Permian of Madagascar and the paraphyly of "younginiform" reptiles. Canadian Journal of Earth Sciences *46*, 651-661. https://doi.org/10.1139/E09-038

7. Marjanović, D., and Laurin, M. (2019). Phylogeny of Paleozoic limbed vertebrates reassessed through revision and expansion of the largest published relevant data matrix. PeerJ *6*, e5565. http://doi.org/10.7287/peerj.preprints.1596v1

8. Müller, J., and Reisz, R. R. (2006). The phylogeny of early eureptiles: comparing parsimony and Bayesian approaches in the investigation of a basal fossil clade. Systematic biology *55*, 503-511. https://doi.org/10.1080/10635150600755396

9. Schoch, R. R. (2013). The evolution of major temnospondyl clades: an inclusive phylogenetic analysis. Journal of Systematic Palaeontology *11*, 673-705. https://doi.org/10.1080/14772019.2012.699006

10. Bapst, D. W. (2012). Paleotree: An R package for paleontological and phylogenetic analyses of evolution. Methods in Ecology and Evolution 3, 803–807. https://doi.org/10.1111/j.2041-210X.2012.00223.x.

11. R Core Team. (2023). R: A language and environment for statistical computing. Vienna, Austria: R Foundation for Statistical Computing. https://www.R-project.org.
